# Pilot study on ^11^C-CFT dynamic imaging using total-body PET/CT: biodistribution and radiation dosimetry in Parkinson's disease

**DOI:** 10.3389/fneur.2023.1153779

**Published:** 2023-05-16

**Authors:** Mei Xin, Lianghua Li, Cheng Wang, Hongda Shao, Jianjun Liu, Chenpeng Zhang

**Affiliations:** Department of Nuclear Medicine, Renji Hospital, Shanghai Jiao Tong University School of Medicine, Shanghai, China

**Keywords:** ^11^C-CFT, dopamine transporter, total-body PET/CT, time-activity curve, effective dose

## Abstract

**Objective:**

Total-body PET/CT equipment, uEXPLORER, is a newly developed imaging technology with a superior resolution, high sensitivity, and high signal-to-noise ratio, providing unique application advantages in the pharmacokinetic evaluation of positron tracers. While ^11^C-CFT PET/CT has been widely utilized in the early diagnosis of Parkinson's disease (PD), it is limited by the short half-life of the radionuclide and an incomplete understanding of its biological distribution in humans. This study aimed to use a total-body PET/CT dynamic scan with ^11^C-CFT imaging to describe the real-time internal biodistribution in PD patients and to obtain accurate radiation dosimetry.

**Methods:**

Six male subjects with suspected PD underwent dynamic ^11^C-CFT total-body PET/CT. Following a bedside intravenous bolus injection of 373.3 ± 71.56 MBq of ^11^C-CFT, PET acquisition was performed synchronously for 75 min with a maximum axial field of view (AFOV) of 194 cm. Time-activity curves (TACs) were generated by delineating volumes of interest (VOIs) of the sourced organs using PMOD software. Tracer kinetics and cumulative organ activities were calculated, and absorbed doses were calculated and estimated using the OLINDA/EXM software.

**Results:**

In the systemic TAC analysis of ^11^C-CFT, several unique types of distribution patterns were obtained among several major organs, including a “Fast-in Fast-out” pattern in the kidneys, lungs, spleen, and thyroid, a “Fast-in Slow-out” curve in the heart wall, a “Slow-in Slow-out” mode in the liver, a “Low-level extending” pattern in the whole brain and muscle, and a “Slow-in to plateau” trend in the striatum and bone. The effective dose of ^11^C-CFT was calculated to be 2.83E-03 mSv/MBq, which is only one-third of the literature value measured by the conventional method. Moreover, this dose is much lower compared to all other doses of DAT radioligands used in PET imaging.

**Conclusion:**

This study is a pioneering application of total-body PET/CT to ^11^C-CFT dynamic imaging. Our results confirmed that ^11^C-CFT has a favorable total body biodistribution, an extremely low internal radiation dose, and high imaging quality, making it suitable for reasonable PD diagnosis in patients requiring multiple follow-up examinations.

## Introduction

Parkinson's disease (PD) is a progressive neurodegenerative disorder that is commonly observed in elderly populations. Despite having been first described two centuries ago, our understanding of the disease has continued to evolve over time (1). The principal pathophysiological characteristic of PD is the degeneration of nigrostriatal neurons and the resultant loss of dopamine, which is responsible for most of the classic motor symptoms, such as bradykinesia, rest tremor, rigidity, and gait instability. Hence, it is crucial to visualize and identify global and regional changes in neurotransmitters related to PD for an accurate diagnosis.

Although dopamine levels cannot be directly measured through imaging, advances in molecular neuroimaging have resulted in the availability of several approaches to mapping the function of dopamine nerve terminals using pre- and post-synaptic ligands (2). Among these tracers, those targeting the dopamine transporter (DAT) in the pre-synaptic membrane are becoming increasingly important as biomarkers for dopaminergic studies. One such representative imaging agent, ^11^C-2-beta-carbomethoxy-3-beta-(4-fluorophenyl) tropane (^11^C-CFT), has been used for early PD diagnosis based on standard PET/CT scans (3–5). However, past approaches using so-called “whole-body” multi-bed and multi-timepoint PET/CT imaging may not accurately access the exact pharmacokinetics of ^11^C-CFT, leading to bias in radiation dose estimates for PD patients.

In this study, we utilized a total-body PET/CT scanner (uEXPLORER, United Imaging Healthcare) to study DAT in a real-time and dynamic mode, enabling evaluation from the “sectional whole body” to the “integral total body.” The large equipment's super-long axial field of view (AFOV) and ultra-high resolution, high sensitivity, and high signal-to-noise ratio allowed us to accurately observe tracer biodistribution throughout the entire human body (6). Time-activity curves (TACs) were assessed to determine dosimetry, which is possible with standard PET/CT but would substantially benefit from total-body PET/CT (7). Studies have shown that dynamic scanning and algorithms using total-body PET/CT can effectively yield a much smaller dose estimate value than the previous method (8). Accurate internal dosimetry is not only important for radiation protection, but also helpful for optimizing injection dose, improving image quality, and streamlining clinical workflow. Therefore, we planned to update and optimize the radiation dose estimates of ^11^C-CFT using the dynamic protocol of total-body PET/CT imaging.

## Materials and methods

### Radiopharmaceutical preparation

The preparation of ^11^C-CO2 radionuclide was performed with the medical cyclotron (HM-10, Sumitomo) at the Department of Nuclear Medicine in Renji Hospital, Shanghai, China. The radionuclide was then synthesized, and quality controlled with high efficiency and radiochemical purity of over 95% by following the previous method in detail using CFN-MPS-200 (Sumitomo) (9).

### Patients

A total of six patients with suspected Parkinson's disease were retrospectively enrolled in this study between January and April 2021. Prior to the PET/CT imaging, we confirmed that none of the participants were allergic to anhydrous ethanol, which was used as the essential solvent in the synthesis of ^11^C-CFT. The retrospective study was approved by the Ethics Committee of Renji Hospital, and written informed consent was waived from the patients. The study complied with the principles of the Declaration of Helsinki.

### Total-body PET/CT scanning protocol

All six patients underwent ^11^C-CFT total-body PET/CT imaging using the digital time-of-flight (TOF) uEXPLORER PET/CT scanner after withdrawing from all PD-related medications for at least 12 h. Prior to scanning, subjects were required to empty their urinary bladders and lie comfortably on the examination mattress in a supine position with arms held side-by-side to ensure optimal positioning and reduce motion artifacts during dynamic acquisitions. CT scans from the vertex to the toes was conducted using a fixed tube voltage of 120 kV for attenuation correction and anatomical localization, along with the auto-mAs technique for dose modulation. A bedside intravenous bolus injection of ^11^C-CFT was administered through a unilateral lower extremity vein, and PET acquisition was launched simultaneously with a maximum axial field of view (AFOV) of 194 cm for a 75-min dynamic PET scan. The raw PET data were reconstructed into 97 frames (24×5 s, 20×30 s, 48×60 s, and 5×180 s) using the standard order subset expectation maximization (OSEM) algorithm with TOF, point-spread function (PSF), three iterations, 20 subsets, 256 × 256 matrix, 600-mm FOV, 2.886-mm slice thickness, and a Gaussian postfilter with a full width at half maximum (FWHM) of 3 mm. The PET image reconstruction process also included scatter and random corrections, dead time, and normalization.

### Time-activity curves

Image processing of the PET/CT scans was performed by an experienced nuclear physician using PMOD 4.0 software (PMOD Technologies LLC, Zurich, Switzerland) (10). The 97 frames were merged into a single dynamic sequence to quantify tracer dynamics using co-registered dynamic PET and CT images. To account for the partial volume effect, this study involved delineating volumes of interest (VOIs), which were found to be 1–3 mm smaller in all dimensions than the actual region of interest observed in the images. The boundary definition of this VOI should be based on either CT or PET imaging, and we prioritized the smaller area as the more accurate representation of the region of interest. In this study, all organs were rendered to scale as previously specified, except for the skeletal bone, which was represented by the proximal femur. Time-activity curves (TACs) were then automatically generated using the kinetic modeling module of the PMOD software while taking into account the radioactive decay. The generated TACs were used to observe changes in uptake by the source organs.

### Radiation absorbed dose estimates

The tracer kinetics and cumulative organ activity of the ^11^C-CFT PET/CT scans were calculated to estimate the absorbed internal radiation dose. The trapezoidal integral over 75 min was employed to obtain the cumulative organ activity (Bq-hr/Bq). Standard organ volumes from OLINDA/EXM 1.0 software (Vanderbilt University, Nashville, TN, USA), as described in a previous study (11), were used to estimate cumulative total organ activity. For each participant, the effective dose and individual organ doses were computed.

## Results

### Overview of the dynamic PET images

**Figure 1:**
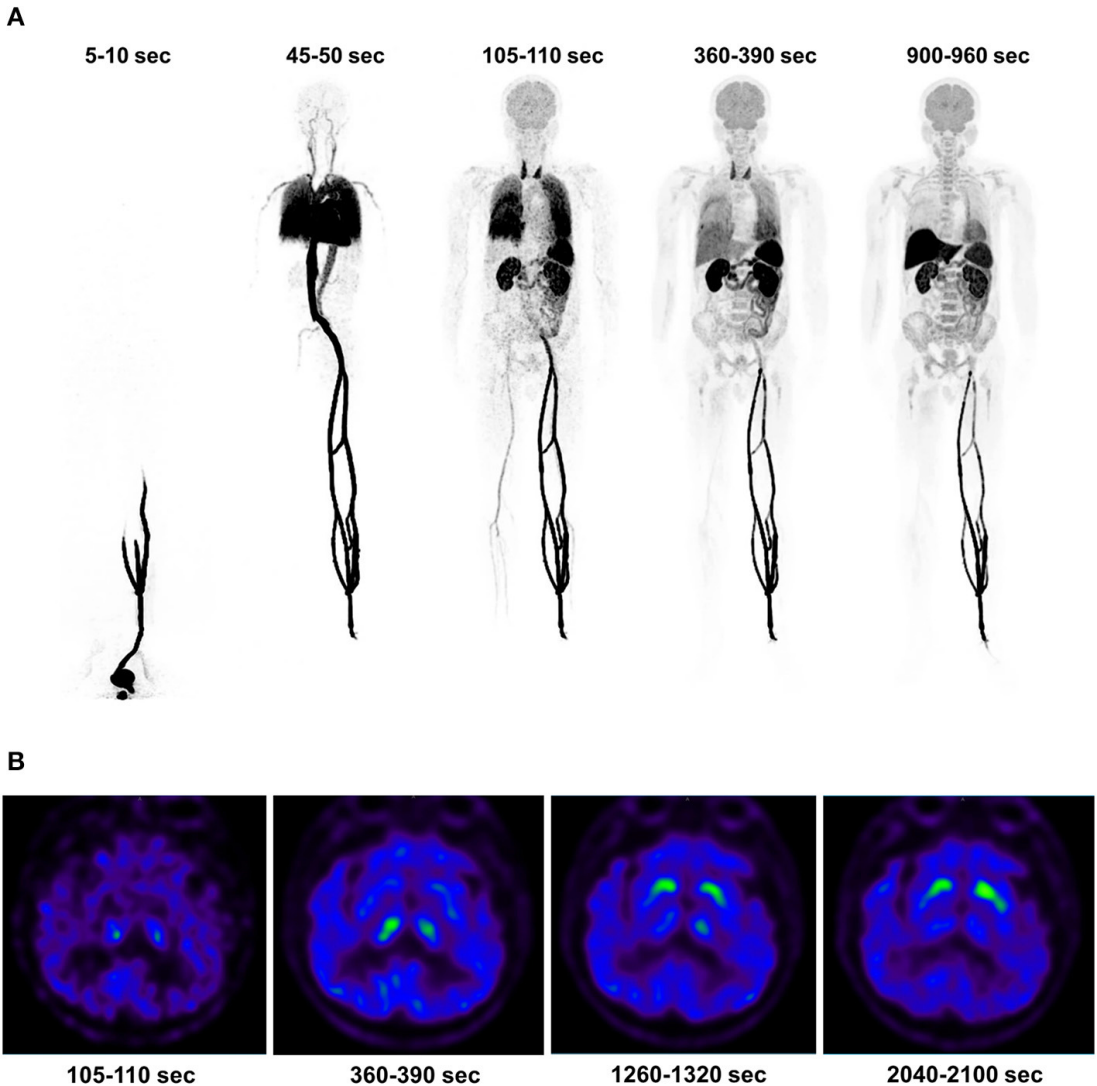
The dynamic PET images of a 73-year-old patient with Parkinson's disease were reconstructed, and the maximum intensity projection of the total body uptake at specific time points was generated **(A)**. First, a duration of 5-10 s indicates the ^11^C-CFT injection and a duration of 45-50 s reflects the distribution in the systemic and pulmonary circulation. A duration of 105-110 s indicates high uptake in the thyroid, while a duration of 360-390 s illustrates the descent of the pulmonary circulation. A duration of 900-960 s indicates high uptake in the liver. Additionally, the cerebral transverse-sectional PET images of the basal ganglia plane displayed the selected time points **(B)**, including a duration of 105-110 s indicating the background uptake of the brain without developing the striatum image. A duration of 360-390 s showed an initial uptake in the striata, followed by a clear outline of the striata at 1,260-1,320 s. Finally, 2,040-2,100 s showed high uptake in the striata.

All six patients received the ^11^C-CFT injection safely and without any immediate discomfort. During the 75-min dynamic scans, decreased uptake of ^11^C-CFT was observed in the striatum of the brains of all participants. Patient information is listed in Table 1. The reconstructions of the PET images obtained at different time points for the total body and the transverse-sectional brain are depicted in Figure 1.

**Table 1:** Patient characteristics (*n* = 6).

**Characteristics**	**Mean ± SD (range)**
Age (years)	59.67 ± 8.89 (48–73)
Height (m)	1.74 ± 0.05 (1.69–1.83)
Weight (kg)	78.83 ± 5.27 (73.00–88.00)
Body mass index (kg/m^2^)	26.03 ± 2.10 (22.99–28.73)
Injected dose (MBq)	373.30 ± 71.56 (264.70–479.20)
Injected dose per kg (MBq/kg)	4.78 ± 1.13 (3.44–6.56)

### TAC studies of the main organs

**Figure 2:**
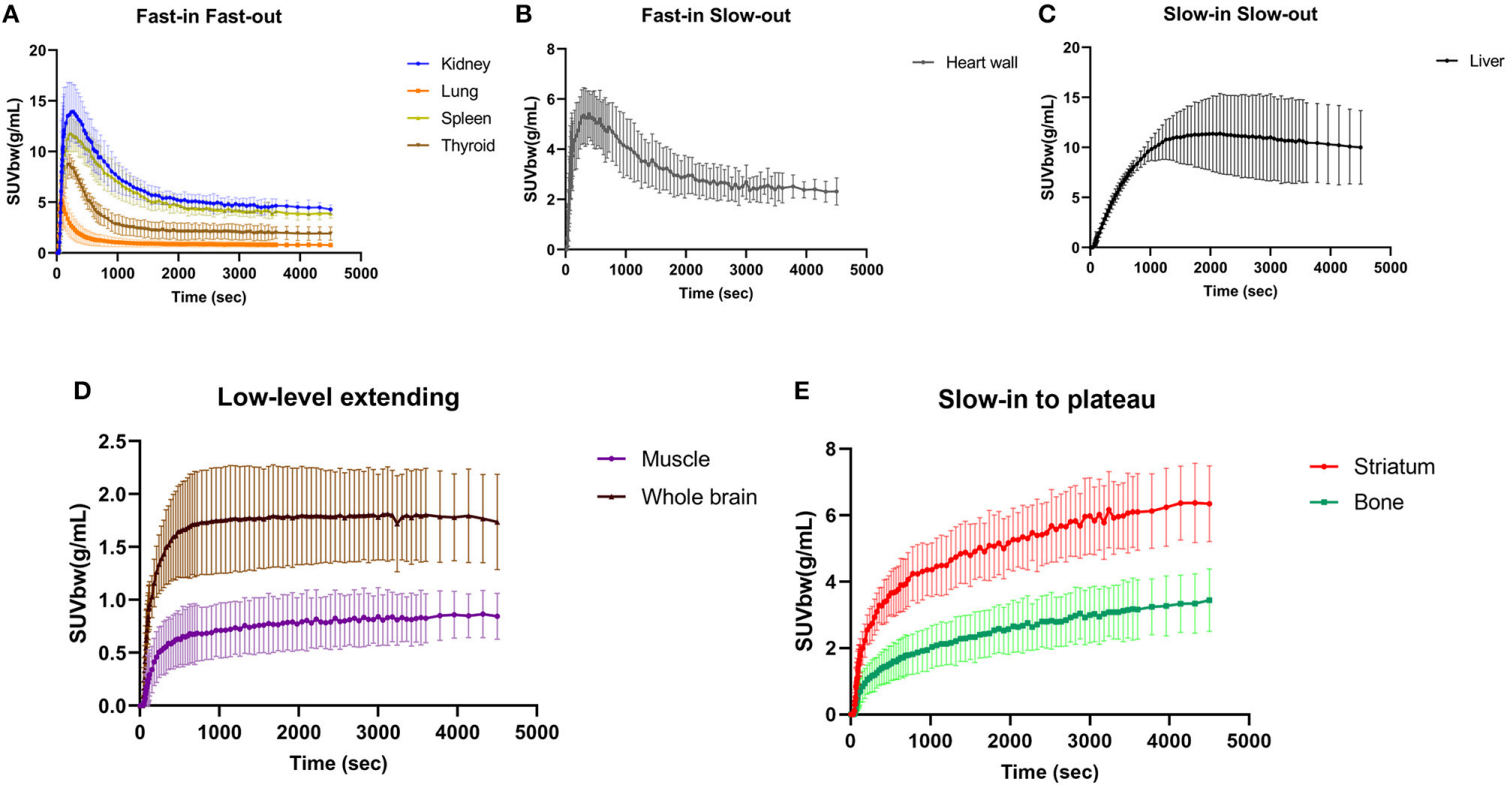
Time-activity curves were generated for major organs, such as the kidney, lung, spleen, and thyroid, as shown in panel **(A)**. Panel **(B)** shows the time-activity curve of the heart wall, while panel **(C)** shows the liver. The time-activity curve for the whole brain and muscle is illustrated in panel **(D)**. Finally, panel **(E)** shows the time-activity curves for the striatum and bone.

The major organs of all six participants exhibited consistent characteristic biodistribution patterns of ^11^C-CFT, as shown by the corresponding TAC analyses presented in Table 2 and Figure 2. Tracer uptake displayed a “Fast-in Fast-out” pattern in the kidney, lung, spleen, and thyroid, with rapid increases and decreases observed (Figure 2A). Specifically, the kidney had the highest mean radioactivity count (SUVbw 14.80 ± 3.11 g/mL), while the lung had the shortest time to reach peak activity (57.50 ± 18.64 sec), followed by the thyroid (189.20 ± 68.59 sec). Spleen and kidney shared a similar time of peak arrival (275.00 ± 79.18 s and 268.30 ± 125.40 s, respectively). The heart wall exhibited a rapid tracer uptake but was followed by a slow decline, characterized as “Fast-in Slow-out” uptake (Figure 2B). The liver displayed a “Slow-in Slow-out” pattern, where the ^11^C-CFT uptake slowly increased until it reached peak activity (1770.00 ± 703.60 sec) and then gradually decreased (Figure 2C). The whole brain and muscle showed generally low degrees of ^11^C-CFT uptake, resulting in a “Low-level extending” curve pattern over time (Figure 2D). The striatum and bone shared a similar TAC pattern, characterized as “Slow-in to plateau,” where the ^11^C-CFT uptake in the striatum slowly increased and finally reached a plateau activity at 3630.00 ± 345.20 sec (Figure 2E).

**Table 2:** Time-activity curve patterns in major organs.

**TAC curve pattern**	**Organ**	**Time of peak or plateau arrival (s)**	**Peak or plateau activity (g/mL)**
Fast-in Fast-out	Kidney	268.30 ± 125.40	14.80 ± 3.11
	Lung	57.50 ± 18.64	6.06 ± 2.04
	Spleen	275.00 ± 79.18	12.14 ± 1.80
	Thyroid	189.20 ± 68.59	9.30 ± 1.82
Fast-in Slow-out	Heart wall	385.00 ± 87.81	5.75 ± 1.16
Slow-in Slow-out	Liver	1770.00 ± 703.60	12.09 ± 3.72
Low-level extending	Whole brain	2270.00 ± 1335.00	1.91 ± 0.50
	Muscle	3540.00 ± 796.90	0.89 ± 0.25
Slow-in to plateau	Striatum	3630.00 ± 345.20	6.58 ± 1.22
	Bone	4240.00 ± 482.50	3.47 ± 0.90

### Radiation dosimetry analysis

To determine the radiation absorbed doses to the target organs, OLINDA/EXM software was utilized. Based on the data presented in Table 3, the liver had the highest radiation absorbed dose (6.40E-04 mSv/MBq), followed by the urinary bladder (3.41E-04 mSv/MBq), lungs (3.25E-04 mSv/MBq), red bone marrow (2.35E-04 mSv/MBq), stomach (2.31E-04 mSv/MBq), kidney (2.28E-04 mSv/MBq), and thyroid (2.24E-04 mSv/MBq). The estimated mean effective dose was 2.83E-03 mSv/MBq. In Table 4, the reference values of radiation dosimetry for different DAT PET imaging agents from previous literature were also provided for comparison.

**Table 3:** Radiation dose estimates for ^11^C-CFT *(mSv/MBq, n* = 6).

**Target organ**	**Mean**	**SD**
Liver	6.40E-04	1.32E-04
Urinary Bladder Wall	3.41E-04	2.61E-04
Lung	3.25E-04	4.64E-05
Red Bone Marrow	2.35E-04	2.18E-05
Stomach Wall	2.31E-04	1.25E-05
Kidney	2.28E-04	2.78E-04
Thyroid	2.24E-04	4.61E-05
^*^LLI Wall	1.92E-04	2.03E-05
Spleen	4.22E-05	5.34E-06
^†^ULI Wall	2.46E-05	4.77E-06
Brain	1.24E-05	4.60E-06
Effective Dose	2.83E-03	2.63E-04

^*^LLI, lower large intestine; ^†^ULI, upper large intestine.

**Table 4:** Effective dose for ^11^C-CFT and other DAT imaging agents.

**Imaging agents**	**Effective dose per injected activity (mSv/MBq)**	**Injected activity (MBq)**	**Effective dose (mSv)**
^11^C-CFT (This work)	2.83E-03	373.30	1.06
^11^C-CFT Huang et al. (20)	8.89E-03	472.06	4.20
^18^F-FPCIT Robeson et al. (21)	1.20E-02	185.00	2.22
^18^F-FE-PE2I Lizana et al. (15)	2.30E-02	200.00	4.60
^11^C-PE2I Ribeiro et al. (22)	6.40E-03	321.00	2.05

## Discussion

The abnormal change of dopamine function in the nigrostriatal nuclei has been identified as a sensitive biomarker for the evaluation of degenerative movement disorders (12). Molecular imaging targeting DAT is an effective approach for early diagnosis and progression assessment of PD in recent studies (13, 14).

Although ^11^C-CFT has been available as a dopaminergic PET tracer for many years, a comprehensive and systematic interpretation of its biological distribution in humans is yet to be established. Prior studies have often used multi-bed and multi-timepoint PET/CT scans to investigate the internal biodistribution and radiation doses of the imaging agent with repeated invasive blood draws (15). However, this imaging procedure is cumbersome, time-consuming, and susceptible to variability in tracer uptake and clearance times in different organs, leading to inaccurate dose estimates, particularly for radionuclides with short half-lives. Conventional PET/CT scans can also miss crucial time points, particularly during the early phase of tracer uptake, which can lead to biased subsequent dose estimation. However, total-body PET can take advantage of the geometric sensitivity gain to scan multiple half-lives after radiotracer administration (7). Notably, there are no previous reports on the use of dynamic total-body PET/CT for ^11^C-CFT imaging in humans. In this study, we employed a 75-min consecutive acquisition task that fully covered three half-lives of ^11^C (t_1/2_ = 20 min), allowing real-time evaluation of DAT biodistribution.

The study's findings demonstrated a consistent and uniform biodistribution of ^11^C-CFT in all participants, with representative curve characteristics observed in the major organs. The kidney, spleen, lungs, and thyroid exhibited a “Fast-in Fast-out” distribution, while the heart wall presented a “Fast-in Slow-out” curve. The muscles and the whole brain displayed a “Low-level extending” trend. Notably, the liver showed a “Slow-in Slow-out” pattern, with a relatively high level of activity retention throughout the scan period. This is due to the liver's physiological control over the removal of peripheral dopamine and its metabolites, effectively limiting access to the systemic circulation of substantial amounts of dopamine released into the portal circulation from the gastrointestinal organs (16). Of particular interest in PD patients, the striatum showed a “Slow-in to plateau” pattern of ^11^C-CFT uptake, with an average time tending to plateau at ~3630 s, which is consistent with the imaging time of static PET scans collected 1 h after ^11^C-CFT injection in our daily work. Interestingly, a TAC pattern similar to that in the striatum was unexpectedly discovered in bone. Bone metabolism may be regulated by the nervous system, and dopamine has been reported to stimulate osteoblast proliferation, differentiation, and mineralization (17). The dopamine transporter gene deletion model has been instrumental in elucidating the specific role of dopamine in bone biology (18). Additionally, a research study has shown that the absence of the DAT gene results in impairments in skeletal structure and integrity (19). Dopamine also affects calcium and phosphorus metabolism. Therefore, the synchronous expression of DAT in bone partly reflects the neuroendocrine functions of peripheral dopamine.

Our study, which utilized dynamic, long-time measured TACs and total-body ^11^C-CFT PET/CT imaging, found that this DAT-targeted agent resulted in an effective dose of 2.83E-03 mSv/MBq in PD patients. Previous radiation dosimetry research on ^11^C-CFT estimated the effective dose per injected activity to be 8.89E-03 mSv/MBq using conventional multi-bed PET/CT scanning techniques (20), which was more than three times higher than the dose estimates from our study. The observed discrepancies in radiation dose estimates between Huang's study and our study may be due to differences in study design, PET/CT scanning protocols, statistical analysis, and dose estimation methods. In particular, the previous study used a multi-bed repeated acquisition protocol to simulate real-time whole-body PET/CT scans, whereas our study employed an advanced total-body PET/CT scanner to perform true real-time dynamic scans of the entire human body. After calibration for differences in injected tracer activity, the actual effective dose in our study was 1.06 mSv, which is even lower than the previously reported effective radiation dose for any comparable radioligands, including ^18^F-FPCIT (1.20E-02 mSv/MBq) (21), ^18^F-FE-PE2I (2.30E-02 mSv/MBq) (15), and ^11^C-PE2I (6.40E-03 mSv/MBq) (22). Although previous studies have indicated that the internal radiation doses of DAT PET tracers are safe and acceptable to the public, our investigation provided substantially lower effective dosimetry for ^11^C-CFT. The technical advantage of dynamic acquisition and measurement by total-body PET/CT facilitated accurate dose estimation, as previously described in the ^18^F-FDG radiation dosimetry study by Hu et al. (8). They have demonstrated that the conventional multi-bed PET/CT scanning method is prone to overestimating the radiation dose to the urinary bladder. Lower radiation exposure coupled with higher image quality will benefit the further application of ^11^C-CFT in visualizing dopaminergic function and evaluating PD and other related movement disorders. In clinical practice, the routine use of administered activities ranging from 259 to 444 MBq (7 to 12 mCi) in a single ^11^C-CFT PET scan would yield effective doses of only 0.73 to 1.26 mSv, which is an extremely low radiation burden and more favorable for patients requiring multiple follow-up examinations. Our study shows that the tracer reaches a plateau within 75 min and remains stable thereafter, indicating that prolonged scan times may not be necessary for clinical diagnosis. However, we acknowledge that this plateau phase may result in dose underestimation. Therefore, a further investigation into dose optimization strategies is warranted. In our daily clinical use of ^11^C-CFT PET/CT imaging, we have not received any feedback concerning toxicity or moderate-to-severe side effects.

Nonetheless, very few patients reported experiencing mild headaches that were transient. Owing to the limited information and duration of the reported headaches, it remains uncertain whether there is a causal relationship between the use of ^11^C-CFT and this observed effect. Further research is warranted to elucidate the underlying mechanisms of such side effects and to devise strategies to optimize patient safety in nuclear medicine practice.

Given the current limitations of our study, our next steps will involve achieving longer scan times using dynamic ^11^C-CFT total-body PET/CT in healthy volunteers who demonstrate good compliance. Additionally, in future investigations, we intend to expand our sample size and carefully consider potential gender-based differences in our study design.

## Conclusion

Using a novel approach with real-time dynamic total-body PET/CT scans, we have conclusively demonstrated that ^11^C-CFT has good tissue biodistribution in various human organ systems with effective internal doses that are lower than previously anticipated. The unique features of low radiation doses and high imaging quality make this agent a viable option for PD patients. With such a safer dose range, PET/CT diagnosis and subsequent repeat examinations may become more feasible and clinically practical for individuals in need.

## Data availability statement

The original contributions presented in the study are included in the article/supplementary material, further inquiries can be directed to the corresponding authors.

## Ethics statement

The studies involving human participants were reviewed and approved by the Shanghai Jiao Tong University School of Medicine, Renji Hospital Ethics Committee. The patients/participants provided their written informed consent to participate in this study. Written informed consent was obtained from the individual(s) for the publication of any potentially identifiable images or data included in this article.

## Author contributions

Conceptualization: MX, CZ, and JL. Methodology: LL and CW. Formal analysis, investigation, and Writing—original draft preparation: LL and MX. Writing—review and editing: MX, HS, CZ, and JL. Acquisition of financial support: CW and JL. Supervision: CZ and JL. All authors contributed to the article and approved the submitted version.

## References

[1] Kalia LV Lang AE. Parkinson's disease. Lancet. (2015) 386:896–912. 10.1016/S0140-6736(14)61393-3 25904081

[2] Strafella AP Bohnen NI Perlmutter JS Eidelberg D Pavese N Van Eimeren T. Molecular imaging to track Parkinson's disease and atypical parkinsonisms: New imaging frontiers. Mov Disord. (2017) 32:181–92. 10.1002/mds.26907 28150432

[3] Zhu S Ju Z Wu P Liu F Ge J Zhang H. The Parkinson's Disease Progression Neuroimaging Initiative. Behav Neurol. (2021) 2021:2230196. 10.1155/2021/2230196 35003386 PMC8739530

[4] Sun X Liu F Liu Q Gai Y Ruan W Wimalarathne DN. Quantitative research of (11)C-CFT and (18)F-FDG PET in Parkinson's disease: a pilot study with neuroq software. Front Neurosci. (2019) 13:299. 10.3389/fnins.2019.00299 31024233 PMC6460224

[5] Liu FT Ge JJ Wu JJ Wu P Ma Y Zuo CT. Clinical, dopaminergic, and metabolic correlations in Parkinson disease: a dual-tracer PET Study. Clin Nucl Med. (2018) 43:562–71. 10.1097/RLU.0000000000002148 29863572

[6] Badawi RD Shi H Hu P Chen S Xu T Price PM. First human imaging studies with the EXPLORER total-body PET scanner. J Nucl Med. (2019) 60:299–303. 10.2967/jnumed.119.226498 30733314 PMC6424228

[7] Nardo L Schmall JP Werner TJ Malogolowkin M Badawi RD Alavi A. Potential roles of total-body PET/computed tomography in pediatric imaging. PET Clin. (2020) 15:271–9. 10.1016/j.cpet.2020.03.009 32498982 PMC8650798

[8] Hu P Lin X Zhuo W Tan H Xie T Liu G. Internal dosimetry in F-18 FDG PET examinations based on long-time-measured organ activities using total-body PET/CT: does it make any difference from a short-time measurement? EJNMMI Physics. (2021) 8:51. 10.1186/s40658-021-00395-2 34264416 PMC8282883

[9] Tang G Tang X Deng H Wang H Wen F Yi C. Efficient preparation of [11C]CH3Br for the labeling of [11C]CH3-containing tracers in positron emission tomography clinical practice. Nucl Med Commun. (2011) 32:466–74. 10.1097/MNM.0b013e3283438f9a 21519304

[10] Sah BR Sommerauer M Mu L Gonzalez GP Geistlich S Treyer V. Radiation dosimetry of [(18)F]-PSS232-a PET radioligand for imaging mGlu5 receptors in humans. EJNMMI Res. (2019) 9:56. 10.1186/s13550-019-0522-9 31240594 PMC6593000

[11] Stabin MG Sparks RB Crowe E. OLINDA/EXM: the second-generation personal computer software for internal dose assessment in nuclear medicine. J Nucl Med. (2005) 46:1023–7. 15937315

[12] Brooks DJ. Molecular imaging of dopamine transporters. Ageing Res Rev. (2016) 30:114–21. 10.1016/j.arr.2015.12.009 26802555

[13] Meles SK Oertel WH Leenders KL. Circuit imaging biomarkers in preclinical and prodromal Parkinson's disease. Mol Med. (2021) 27:1–18. 10.1186/s10020-021-00327-x 34530732 PMC8447708

[14] Brucke T Brucke C. Dopamine transporter (DAT) imaging in Parkinson's disease and related disorders. J Neural Transm. (2021) 15:1–4. 10.1007/s00702-021-02452-7 34910248

[15] Lizana H Johansson L Axelsson J Larsson A Ogren M Linder J. Whole-body biodistribution and dosimetry of the dopamine transporter radioligand (18)F-FE-PE2I in human subjects. J Nucl Med. (2018) 59:1275–80. 10.2967/jnumed.117.197186 29348315

[16] Eisenhofer G. The role of neuronal and extraneuronal plasma membrane transporters in the inactivation of peripheral catecholamines. Pharmacol Ther. (2001) 91:35–62. 10.1016/S0163-7258(01)00144-9 11707293

[17] Lee DJ Tseng HC Wong SW Wang Z Deng M Ko CC. Dopaminergic effects on in vitro osteogenesis. Bone Res. (2015) 3:15020. 10.1038/boneres.2015.20 26558139 PMC4639997

[18] Bliziotes M McLoughlin S Gunness M Fumagalli F Jones SR Caron MG. Bone histomorphometric and biomechanical abnormalities in mice homozygous for deletion of the dopamine transporter gene. Bone. (2000) 26:15–9. 10.1016/S8756-3282(99)00232-X 10617152

[19] Bliziotes M Gunness M Eshleman A Wiren K. The role of dopamine and serotonin in regulating bone mass and strength: studies on dopamine and serotonin transporter null mice. J Musculoskelet Neuronal Interact. (2002) 2:291–5. 15758457

[20] Huang T Wang H Tang G Liang X Deng H Yi C. Human radiation dose estimation of (11)C-CFT using whole-body PET. Clin Nucl Med. (2012) 37:1159–62. 10.1097/RLU.0b013e318266cd1b 23154473

[21] Robeson W Dhawan V Belakhlef A Ma Y Pillai V Chaly T. Dosimetry of the dopamine transporter radioligand 18F-FPCIT in human subjects. J Nucl Med. (2003) 44:961–6. 12791826

[22] Ribeiro MJ Ricard M Lievre MA Bourgeois S Emond P Gervais P. Whole-body distribution and radiation dosimetry of the dopamine transporter radioligand [(11)C]PE2I in healthy volunteers. Nucl Med Biol. (2007) 34:465–70. 10.1016/j.nucmedbio.2007.02.005 17499737

